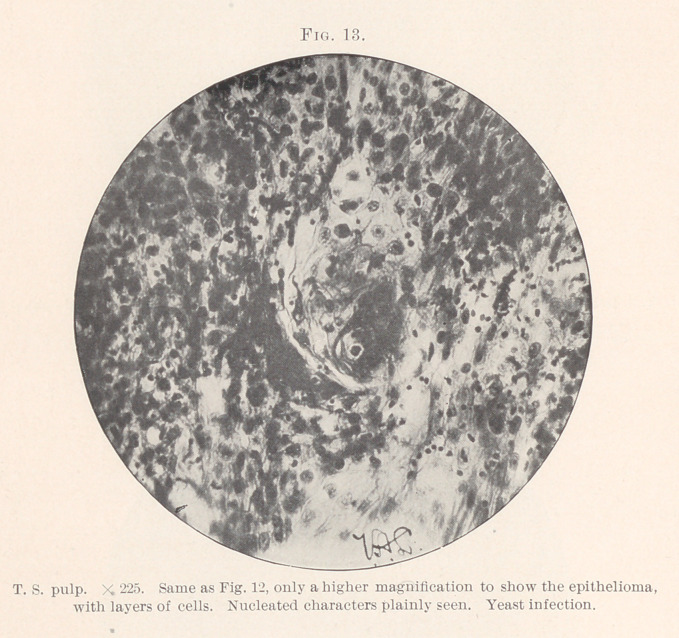# Neoplasm (Epithelioma) of the Pulp

**Published:** 1904-11

**Authors:** V. A. Latham

**Affiliations:** Rogers Park, Ill.


### International Dental Journal.

Vol. XXV. November, 1904. No. 11.

### Original Communications.[1]

### NEOPLASM (EPITHELIOMA) OF THE PULP.[2]

BY V. A. LATHAM, M.D., D.D.S., F.R.M.S., ROGERS PARK, ILL.

The great importance of this subject from a clinical as well as a purely pathologic point of view must be my excuse for presenting this rather incomplete study. It is with diffidence that I put the case on record, for in looking through the literature at my command I have so far been unable to locate any other like condition. If we exclude the hypertrophies which have been described under the heading of "Polypus of the Pulp or Tooth," then I am led to the supposition that this may be the first case recorded of an exceedingly rare condition. In reviewing the literature, Ziegler[3] speaks of "tumors of dental tissue formed in later life, and described by dental pathologists as odontinoids."[4] Accordingly as they consist of enamel, dentine, cement, or a combination of these, they are classified as enameloid, enamelodentinoid, dentinoid, den-

[1] The editor and publishers are not responsible for the views of authors of papers published in this department, nor for any claim to novelty, or otherwise, that may be made by them. No papers will be received for this department that have appeared in any other journal published in the country.

[2] Read at the fifty-fifth annual session of the American Medical Association, in the Section on Stomatology, Atlantic City, June 7 to 10, 1904.

[3] Ziegler, Special Pathological Anatomy, section ix. p. 593, 1897.

[4] Ullrich, Ueber feste Neubildungen in der Zahnhöhle, 1852.

tino-osteoid, or osteoid.[1] They are all small, often to be recognized only by the aid of the magnifying lens. They are flat, round, pear-shaped, or warty in appearance. The first three-named varieties grow from odontoblasts, and arise from the pulp of the crown and from that of the root, generally in connection with caries, under metallic fillings, or as a result of periostitis, mechanical injuries, abnormal retention of teeth, or senile degeneration. The osteoid form grows from the pulp and from the periosteum, and is developed from osteoblasts. "Sarcoma, fibroma, and myxoma are in rare cases developed from the pulp as the tooth is being formed. Such growths, and particularly the sarcomata and fibromata, are, however, much more commonly derived from the periosteum of the dental socket or alveolar process, from the bone marrow or from the gum itself" (Ziegler).

Falkson[2] has described cases of cystadenoma arising from the rudimentary tooth papillæ and taking the form of a multilocular cyst, produced by cystic degeneration of the dental follicles. The growth encloses newly formed gland-like tubules and acini.

P. Bruns[3] and others have recorded certain rare instances of dental carcinoma, in which some of the epithelial cells of the tumor take on the appearance of enamel germs and produce enamel.[4] Unfortunately, the term "sarcoma" has been used when a simple hypertrophy or polypus of the pulp has been meant, and this has been passed down from Wedl[5] and Salter[6] to Black.[7]

Bödecker[8] states that the only known tumor of the pulp is malignant myeloma, as first described by C. Wedl. Bödecker adds

---

[1] Schlenker, Zahn- und Mundpflege, St. Gallen, 1883; Untersch. über das Wesen der Zahn-verderbniss, St. Gallen, 1882; Pulpen Odontinoide, Hdbuch d. Zahnheilkunde; Verknöcherung. der Zahnnerven, 1885; Vierteljahrsschrift f. 3, Wien, 1892.

[2] Falkson, Development of Rudimentary Teeth and Cysts of the Jaw, V. A. 76, 1879.

[3] Bruns, P., and Chibert, Adamantine Epithelioma. A. de méd. exp., 1894, with references.

[4] Mallassez, Epithelial Detritus round the Roots of Teeth in Adults. A. de Physiologie, v., 1885. Massin, Congenital Epithelioma originating from the Enamel, V. A. 136, 1894.

[5] Wedl, Pathologie der Zähne, 1870. Transl.

[6] Salter, Dental Surgery and Pathology, 1874.

[7] Black, American System of Dentistry, p. 915.

[8] Bödecker, Anatomy and Pathology of the Teeth, 1894.

one other example, but unfortunately nothing was known concerning the history of the pulps affected.

In both cases, as seen by microscopic examination, they were the so-called round sarcoma, or, as Bödecker prefers to call them, lymphomyeloma. The term myeloma is used synonymously with sarcoma, some authorities objecting to sarcoma on account of its derivation from the word "sarkos" (Greek, flesh), whereas myeloma means what these tumors in reality are, medullary tumors. When combined with epithelial as well as connective tissues, we must not forget that they are termed "medullary carcinoma," and in some cases may lead to a wrong diagnosis.

Dr. Juan M. Alberdi,[1] Madrid, says, under affections of the dental pulp, "The organic affections consist in tumors, which reside in this organ. The tumors are hypertrophied productions, brought about by the simple hypergenesis of the normal elements of the tissue." The tumors are always the consequence of some lesion, especially penetrating caries, with exposure of the pulp. They are smooth, of a grayish color, and sometimes of considerable size, covered by a pellicle, which is only a thickening of that which covers the normal pulp. The treatment consists in removal of the pulp.

Dr. A. Pont[2] (de Lyon) mentions a case of tumor of the dental pulp without caries, which is not a tumor, pathologically speaking, but a polypus, or primary hypertrophied pulp with secondary inflammation, without power to form secondary dentine, but which has absorbed the ivory dentine, which properly belongs to the tumor division, as seen so often in the osteosarcomata, and might incline one to place it in this group. "To what it was due is not clear, as it could not have been a chronic or hypertrophic pulpitis, there being no history of pain or caries. It might have been an aneurism of the pulp, but absence of hemorrhage excludes that, hence we will call it an hypertrophy of pulp, with total absorption of the dentine without caries."

When we review the histology and gross anatomy of the pulp, and note its close relation with the connective-tissue origin, we

---

[1] Alberdi, Dental Cosmos, p. 916, 1902, Third International Dental Congress.

[2] Pont, A., L'Odontologie, September 15, 1902; Dental Cosmos, November, 1902, Transl.; Ash's Quarterly Circular, December, 1902; Second report, with microscopic findings, in L'Odontologie, May 30, 1903.

easily see its liability to almost every known disease. Its peculiar formation enables it to supply the nutritive needs of the tooth with the vascular and nervous endowments, in which it is particularly rich.

The study of pulp diseases is best made by obtaining the affected teeth immediately after they are extracted. Great care is necessary that an observer be not led by his preconceptions. Just opinions are arrived at only after a thorough examination, and by comparison with many other similar cases. Diseased conditions of the pulp are to be studied in the pathologic associations of inflammation, trauma, and new formations.

Albrecht, in his monograph on "Diseases of the Pulp," follows these divisions: (*a*) disease of nerve; (*b*) disease of blood-vessels; (*c*) disease of secretion. To-day this will avail nothing, as it is founded on no anatomic pathologic observation, and only confuses by the intermixture of terms which is bound to exist.

When we consider the position occupied by the teeth at the entrance of the alimentary tract, the change of temperature, varieties of food, continual changes in blood-pressure as a peripheral area, and (in my mind one of the most important conditions to explain pathologic changes in their structure) liability to faulty development, fissures, cracks through acidity, abrasion, erosion, caries, and almost sure entrance of bacteria, as well as the relation and condition of surrounding structures as gingivitis, and the close effect of constitutional and systemic conditions of health, is it any wonder we have hyperæmia, which may readily be followed by more or less pulpitis, the quick follower of any irritation? Not always does it happen, however, that pain does follow pulp exposure of the peripheral tubuli; even when the inner aspect of the tubes is seen in a hyperæmic state, the pulp is often found in a fair state of resistance. The conductors of irritation, the odontoblasts, are common in their relationship to tubules and central organs; the filling up of these tubes and shrinkage of these cells break up the line of conduction, and this constitutes self-protection; then secondary deposits occur, and less pain usually follows. If the condition progresses, we may have a pulp exposure, with all the signs and symptoms of pulpitis. Pain is not necessarily an associate of the inflammation.

When a pulpitis has been of long continuance the pain seldom is found restricted to the tooth, but is apt to diffuse over all the trigeminal tract. Sympathetic neuralgias are not the sequences alone of pulpitis, one of the commonest causes being erosion just under the gingival margin.

Baume[1] considers pulpitis is rare in children, and only occurs when caries has attacked the organs before absorption begins.

There are many sequelæ of inflammation which bear on the etiology of neoplasms: 1. Chronic pulpitis, which may even be the primary condition. 2. Suppuration. 3. Hypertrophy. 4. Gangrene. 5. Periodontitis. 6. New formations. In chronic pulpitis we have little to distinguish between such as are inflamed or not, death of the pulp following very rapidly in some cases. There is no vital resistance, but an unresisting, decomposing, greasy mass, which easily explains the loss of pain during death of the pulp.

*Suppuration.*—This is indicated by a sense of fulness, uneasiness, and weight, and sometimes pulsation or boring pain, which may be of slight degree in some cases, but may be accompanied by œdema in and about the face in others. This is one of the commonest forms of termination of chronic pulpitis. Scanty watery pus, or pus of an acrid nature, is often met with where we have some constitutional disease, or where disease of the overlying parts has preceded or caused suppuration. When pus is sealed in a cavity for any length of time, without external agencies, we find it very much thicker, pasty, and with a vile odor of putrescence; microscopically it is very rich in fat.

Gangrene may rapidly follow suppuration, the nutritional cells becoming pus. The conditions due to congestion have been followed, and gangrene succeeds the high tension point of hyperæmia, the breaking up of the cell walls, passage and transformation of the cells, the coloring of the tooth, and the tissues melting down surely, if slowly, until all are destroyed; fat drops running in all directions, and even covering all the nerves and vessels, and ending in a dark-colored, greasy, putrid mass,—sphacelus, or tooth discoloration. Dry gangrene is meant when evaporation of the circulatory secretions of the tooth has occurred.

*Hypertrophy.*—Inflammation may also cause a numerical increase in its constituents (hyperplasia), as well as hypertrophy, and form an enlarged mass so often and erroneously called a tumor. True, the increase in the new growth is allied to the histologic

---

[1] Dr. R. Baume, Lehrbuch der Zahnheilkunde.

structure in its cell elements and becomes a polypus resembling gum tissue, only projecting from the *interior* of the pulp-chamber. Microscopically, we see connective tissue, the vascularity being a characteristic feature. The nerve element being wanting, sensibility is often lacking—*unempfindlich.* Growth is rapid and often recurring readily, resembling malignant growths.

Salter speaks of a "sensitive sprouting of the pulp," differing very little from the above, except that it is clearer and softer and its vitality implies a more abundant nerve supply. It is often relieved only by extraction of the tooth.

Römer [1] has reported an examination of thirty patients with polypus, and gives three layers of granulation tissue: 1. Outer layer of pus cells. 2. Wider zone of endothelial cells, and of capillaries with the appearance of gland strata. 3. Strong tissue, with enlarged blood-vessels and containing many round cells.

When the teeth are extracted they are placed in ten per cent. formalin and decalcified in thirty-three and one-third per cent. solution of formic acid. They are cut in half with a razor and the part wanted embedded in celloidin and stained by any dye, as alum-hæmatoxylin, and picrofuchsin (Van Giessen), or by Weigert's method.

In the head of the polyp no nerve-fibres were seen, but in pulp-chambers and root-canals were nerve bundles, varying according to the degeneration found, which consisted usually of fibroid elements seen best in teeth whose pulps have been exposed by fracturing the tooth in faulty extraction. In the case under consideration there was little to note as being unusual.

The patient, an elderly woman, aged fifty-six, stout build, neurotic type, with signs of bronchial irritation and asthma (two sisters having died of cancer), called to see me regarding a partial plate. After examination, I advised that the few remaining teeth be extracted, as she gave a history of continued neuralgia around the face and head. The teeth were scattered in the mouth, and only the right upper first premolar and cuspid seemed peculiar, the former having a queer reddish color, no carious spots or other evidences of decay. The mouth was very cleanly kept; slight irritation of the gingivæ around the teeth and a feeling of un-

[1] Rümer, Ueber Pulpa Polypen der Zähne. Corresp. Blätt. f. Zahnärzte, C. Ash, Berlin, January, 1892.

easiness about the premolar. On percussing, I found that the nerve was not dead, but evidently congested. On extraction, hemorrhage was free and the tooth seemed to give way reluctantly, with a feeling of pulling from the apical attachment (hyperplasia cementum). I dropped it at once in warm saline solution, and then extracted the cuspid, which was an oddly shaped tooth, of a queer greenish-white tinge, so noticeable that the patient many times said people asked what was the matter with it. The cuspid was exceedingly difficult to remove, and on its coming out a drachm of yellowish clear fluid oozed from the alveolus. The apex of the root was covered with a thickened layer of peridental tissue, and I wondered if it was an abscess sac that had ruptured; the foramen showed a whitish thread hanging as the pulp, and I saw no evidences of suppuration, but some hypercementosis. So I preserved it in salt solution. The socket I carefully cleansed with peroxide of hydrogen; there was no effervescence to speak of, except when bleeding. Then I probed the cavity, and finally curetted it as thoroughly as I could, applied tincture of iodine, and then ninety-five per cent. carbolic acid, and plugged with gauze and wool. This I did for nearly a week, and then allowed the cavity to close. It healed steadily on the slow and gradual withdrawal day by day of the gauze tampon. The patient so far (after three years) has had no recurrence nor trouble, and seems much more comfortable, using no upper plate yet, as she prefers to wait.

The teeth were removed from the saline solution and carefully opened, one longitudinally, and the cuspid transversely about halfway, and the root longitudinally under normal fluid, till appearances were noted, and then it was changed to Zenker's solution. The cuspid had a fairly solid pulp, whitish-green. It looked like a fibroid, and was springy to the touch like a myxoma. I made microsections, and reproduce some photomicrographs from them. Taking the pulp at different levels, we find it complex and offering an unusual appearance.

Several points are worthy of note (Figs. 1 and 2): 1. Evidence of granulation tissue and pulpitis. 2. Slight sclerosis or fibrosis. 3. Slight hyaline degeneration in this area. 4. Small vessels surrounded by fibrous thickening. 5. Many polynuclear leucocytes were scattered throughout, in some places massing together like a round-celled infiltration and mixed in with bands of fibrous trabeculæ. (Figs. 2, 3, and 4.) 6. Near the outer edge were more deeply stained cells and better formed fibrous tissue, which gave a more homogeneous appearance to the cells, and at one side was a dark bluish irregular body, a calcospherite or pulp-stone, longer than it was wide, and with a poorly stained area around it. (Fig. 1.) At one edge of the specimen was an almost glistening hyaline mass of cells, inflammatory, and with some cells, much larger than others and multinucleated, lining the fibrous stroma spaces, almost like scirrhus cancer. (Figs. 5 and 6.) Some of these cells were massed in cluster-nests, showing signs of considerable pressure; some cells staining a pale hyaline pinkish color, with the nuclei taking a darker stain, like cartilaginous cells, others appearing horny and taking no stain. A number of dyes were used, prominently hematoxylin and eosin, Van Giessen, and Ranvier's picrocarmine and logwood, picrofuchsin and nigrosin, safranin, Unna's polychrome-methyl blue.

A method that will be found very useful for gum tissue, polypi, and epithelial structures is one modified and suggested by W. R. Smith, M.B.: Stain the section on the slide with hematoxylin and alum carmine, wash with water and put on the slide; dehydrate with absolute alcohol, stain a few seconds with a saturated solution of saffranin in aniline oil water, of which most is removed with blotting-paper, run over the section absolute alcohol for a few seconds, and immediately drop on clove oil; mount in Canada balsam in xylol or chloroform. Great care is required in the washing out; it must only be partial. Too little alcohol in the washing leaves the stain in the normal tissues, and does not differentiate the cell-nests from these or from other epithelial structures. Too much alcohol removes the whole of the stain from all pathologic and normal tissues. If sections are cut fresh, it is much harder to stain with hematoxylin and to dehydrate. For fresh sections, soak a few minutes in four per cent. formalin or methyl alcohol and then use alum carmine and wash thoroughly in water, and dehydrate. The carmine stains faster if gently warmed. In studying these specimens we must clearly note:

1. Whether normal non-pathologic growths of epithelium in the form of processes are growing inward from an epithelial surface,—*e.g.*, gum, epidermis, mucous membrane, pulp, or glandular organ which can resemble an epithelial pearl when cut in some ways.

2. Pathologic processes, papillary prominences, epithelial columns of homogeneous masses of epithelial cells with ovoid nuclei (papillary prominences of Lebert), which may be found in the surrounding tissue, and simulate cell-nests. Neither of these correspond with Billroth's classical description of cell-nests. What distinguishes these masses in their earliest known form? (Figs. 7 and 8.)

They appear at first sight like spherical or oval cysts from one one-hundredth to one five-hundredth of an inch in diameter, walled in by irregular fibrous tissue, and containing granular matter, nuclei, or cells within them. They may be in cluster or cylindrical; their nuclei are shrivelled or not visible; contents often granular.

The termination in horny material is an extreme form of specialization, and such is not the destiny of all cell-nests. Indeed, the study of these extreme forms is much desired, for observers have thus been misled as to the true nature of the structure and mode of growth of cell-nests in general.[1] (Fig. 13.) In all probability epithelial cell-nests start from one cell, by division and subdivision, except in such a disease with large pearls, as seen by Hamilton.[2]

In Figures 7 and 8 we find a single cell with two nuclei standing out; around it various-sized cells are massed, forming an early cluster, and surrounded by one outer layer of cells; in places, only a single cell and small cohorts of a few cells each. In some places we find here and there a thickened capillary or arteriole, showing well-marked cells lining the endothelium (Fig. 7); here and there a large cell and nucleus, or an alveolus filled with a cluster of cells, almost forming an island. (Fig. 10.)

We find masses like those just described mixed in with the small-celled infiltration of Virchow, indicating active proliferation and irritation, with the intermediate areas breaking down or staining only poorly, but with here and there well-marked nucleated cells. Lying near these masses we see some typical cell-nests with granular detritus among them, singly and in groups. How these cell-nests grow is yet to be understood. Paget[3] is in favor of the

---

[1] Woodhead's Pathology, second edition, 1885, p. 481; third edition, 1892, p. 174.

[2] Text-book of Pathology, 1889, part 1, p. 406.

[3] Paget, Lectures on Surgical Pathology, third edition, 1870, p. 720.

theory of the growth being from one central cell, and Snow,[1] who, with others, gives us a theory of surrounding pressure, says "a small area of cells always appears as the original point of the globe, never a single cell." (Figs. 9, 10, 12.)

That this specimen is a carcinoma, epithelioma, is very evident. It certainly cannot be classed, like Wedl's and Bödecker's cases, as a lymphomyeloma or round-celled sarcoma, for it certainly possesses epithelial nests, fibrous trabeculæ, and stroma, lined with large cells; granulation cells of the polynuclear type, degeneration, and a reaction to dyes that marks the carcinoma group. (Figs. 6, 7, 11, 12, 13.)

In polypus of the pulp the parenchymatous connective tissue is the seat of the proliferation described as sarcoma of the pulp, in which the parenchyma is gradually destroyed, and indicated by the absence of nerves and the altered character of the blood-vessels. As the sarcoma is located on the outside of the remains of the pulp, it serves in a measure to protect the latter.[2]

The study of the odontoblasts is a very vexatious one, as on account of their close relation to the dentine it is difficult to procure good sections without decalcifying the hard tissues, or preparing a tooth by the Koch-Weil process, which loses much valuable material; and the disarrangement of the pulp, unless this is done, renders it valueless.

In removing the pulp from its chamber the layer of odontoblasts clings tenaciously to the dentinal walls, either partly or as a whole, and hence we can easily understand how little progress has been made in this study. Black[3] states that this layer of cells seems to remain unchanged in acute inflammation until combined with suppuration. This seems, judging from my own work, to be hardly the case, for the cells are seen in every state of disease, and many times are not present, showing plainly the odontoblast cells act very similarly to the ciliated cells in bronchitis, and the falling off before the pulp is very much affected shows how easily any irritant affects the deep pulpal ends of the cells, and so causes atrophy and degeneration. (Figs. 4 and 5.) Hopewell-Smith[4]

---

[1] Snow, Cancer and Cancer Processes, London, p. 65.

[2] Wedl, Pathologie der Zähne, 1870. Transl.

[3] Black, American System of Dentistry, p. 915.

[4] A. Hopewell-Smith, The Histology and Pathological Histology of the Teeth, 1903.

also shows illustrations which correspond to my own slides of inflamed odontoblasts, showing in some a numerical hyperplasia. It would be an interesting study to make careful sections of pulps *in situ* and extracted from teeth excised in cases of neoplasms of the jaw, as myeloid sarcoma, epithelial growths, fibroma, epulis, etc., and so note if the pulp shows any metastatic deposits or any other pathologic conditions. I have a few specimens so made which exhibit many problems for elucidation, and especially as regards their origin.

In the specimen here presented it may be asked how and from what did this growth originate. The teeth were, to all appearance, sound; even with an aplanatic lens nothing could be seen but the change in color, which gave me the idea of a dead tooth, and the percussion note was negative. The sensitiveness and neuralgic pain pointed out pulp and peridental irritation. Stanley Colyer[1] shows a "burrowing epithelioma from the periodontal membrane" which illustrates my section of neoplasm of the pulp very well, but he found the pulp dead and suppurating. His theory as to origin would certainly be questioned by many of the workers in carcinoma to-day. (Figs. 11 and 13.)

If we accept the theory that "like cells produce like," then how did odontoblasts produce epithelial cells, if the former originate from the stomodeal mesoblast,—*i.e.*, from the periphery of the dentinal or pulp organ? If odontoblasts could be proved as arising from the epiblast or hypoblast, many difficulties would be removed. Their nerve-endings and their close relationship to the cells at the terminations of the optic and auditory or sensory nerves, not only as regards the physiology, but the morphology and pathology, their functions being allied somewhat to the glandular organs, as in the stomach and intestine,—namely, secretion, excretion, and manufacture of chemical problems for metabolism,—and the situation and better development of odontoblasts at the coronal cervical part show that we have here a close relationship of epiblast and hypoblast structures. This points to the nerve-impressions passing through the tubules, and shows a closer relationship to the nervous system than is usually given.

In looking over the question of treatment we must note the

---

[1] Stanley Colyer, Transactions Odontological Society of Great Britain, June, 1901, pp. 231, 242.

use of the cautery, escharotics, and iodine. In this instance the best and only treatment was extraction, and if it had not been for the usual plan of study by breaking open the teeth and making sections, the great danger the patient ran would never have been recognized, and the chance of continual watching afterwards, as well as the curetting which was done, would have been lost. It is well known that arsenic has been accredited with causing epithelioma, and J. Hutchinson[1] considers that many of the cases of multiple cutaneous sarcomata may be fairly attributed to the use of iodine and its salts. When we note the frequency with which iodine is used in dentistry, externally and internally, that many patent medicines contain it, and consider its use in the arts, photography especially, and in the preparation of antiseptic gauzes, we can find another point for study. The frequent stimulation used by dentists for pericemental and periodontal irritation should cause an increase in hypertrophy, gingivitis, and the papilloma and epulis forms of growth, to say nothing of the epitheliomata; but I think it can be shown we have less neoplasms in the oral cavity now than formerly, due, possibly, to better hygiene and methods of operating.

### CONCLUSIONS.

The points of interest are:

1. The freedom from urgent symptoms beyond neuralgia.
2. The great rarity of neoplasms of the pulp.
3. Lack of literature and careful pathologic and histologic study.
4. The want of further and more complete examination of the tooth-structures, in relation to the pathology of neoplasms of the jaw.
5. The question of metastases in the pulp from periodontal tumors.
6. The value of microscopic study should be urged in dental schools, as an aid to differential diagnosis and treatment.
7. The changes which may attend the killing of pulps by irritants and care to thoroughly cleanse the pulp-canals.
8. To emphasize the better treatment and value of closing up fistulous openings in the gingivæ for fear of further infection, and

---

[1] Archives of Surgery.

irritation of the peridental membrane, which may cause the growth of neoplasms.

9. That all dead teeth should be discovered early and treated thoroughly with the best of surgical skill.

10. The frequency with which hypercementosis is found at the radical point of the dead tooth.

11. The embryonic origin of the various structures of the pulp.

12. That great care should be used when examining the dental structures in elderly people, and all causes of irritation removed.

13. From what did this epithelioma originate, since it is a primary growth, enamel or odontoblasts—most probably the nerve elements? [1]

DISCUSSION ON PAPERS BY DRS. ANDREWS, TALBOT, AND LATHAM.

*Dr. M. L. Rhein,* New York City.—The deductions which Dr. Andrews has made I do not think are logical or warranted by what he has presented to our Section in the last few years. I have never taken the position attributed to me of believing that pulps were merely for formative purpose, and that in adult life they would be better out than in. I do radically disagree with the argument laid down by Professor Andrews that we are too prone to remove diseased pulps, and I believe Dr. Latham's conclusions show the error of conservative treatment of diseased pulps. One of the unfortunate things that meets the practising dentist is the impossibility of making microscopic examination of pulps in a pathologic condition. We can only draw inferences and conclusions. There has come into practice in my locality in the last few years a pulp-capping imported from Germany, known as "iodoformagin." I have a large collection of radiographs taken from one to three years after this medium has been used, and in every instance macroscopic examination has shown an apparent degeneration of pulp-tissue. It has been impossible to make microscopic examinations. In some cases the entire contents of the pulp-canal have apparently been consolidated, so that, so far as a circulating medium, or pulp proper, it has disappeared up to within a close border of the end

[1] Other references which may be consulted are: Latham, Résumé of the Histology of the Pulp, Journal American Medical Association, July 12, 1902; New York Medical Journal, May 10, 1902; also American System of Dentistry, vol. i. p. 883.

of the root. It is for this purpose that I have been for years a strong advocate of the aseptic removal of every portion of the contents, not only of the pulp-chamber, but the canals where this is liable to occur, or where it is feared. The criticism made by Professor Andrews in his paper as to the results of removing the pulp and the subsequent deterioration of the cementum and pericementum tissue is one that I naturally disagree with, and the basis of that disagreement is the observation of following this practice for over twenty years. I do say that there is a vast difference in the manner and methods of removing pulp-tissue and of taking care of those tissues afterwards. The trouble is that the majority of the profession are not willing to give the time required to thoroughly remove every portion of the contents of the pulp. There is another procedure necessary after every portion of the pulp contents has been removed surgically; the introduction of proper therapeutic agents will remove so much of those fibrils that enter the canalicular portion of the dentine as to leave that tooth absolutely free from any danger of breaking down and infection. These agents should be followed by scientific sterilization of the canals. Finally, the hermetical sealing of those canals is one of the means which will keep the roots in healthy condition. Where this healthy condition does not continue, it is, in the majority of cases, due to faulty operation. There is no excuse for a darkened tooth-substance after the removal of tooth-tissue.

*Dr. E. A. Bogue,* New York.—Dr. Rhein has called attention to my misunderstanding of his position. I am glad to have him set right the question of removing a living pulp. He has just acknowledged what I wished to have him,—that a living pulp cannot always be removed instrumentally. This morning he said that he had been misinterpreted and made to say that he considered the pulp of no value after adult life had been reached, and it might better be removed than not. That was not my understanding, but I understand him to publish this statement, that after adult life is reached he regards the pulp, being a formative organ of the tooth, as of little further value. I agree with him in regard to preserving a pulp exposed by decay. I have never seen any capping or treatment of any kind effectual in restoring health, or a condition that would lead to restored health, in these pulps, once exposed. Dr. Rhein told me on one occasion that he had removed a living pulp from a right lower molar. I have that tooth in my possession to-day. It and I do not agree with them. I surely do not mean to remove the pulp. When brought to me, it had considerable of the pulp in it, showing that his skill was not sufficient to get it out; nor could any one else have done it.

*Dr. E. C. Briggs,* Boston.—I take the ground which I have taken before, which is that after adult life the pulp is more often a menace to the tooth than a help. That does not mean that every tooth should have the pulp killed. I think that after the able papers by Drs. Latham and Talbot we are appalled at the ills the flesh is heir to, and it makes one more convinced than ever that the pulp had better be out of the way. The surgical removal of the pulp is the thing that these men do not seem to consider. We know Dr. Andrews's ability and power of research, which I bow to with great respect; yet his deductions are not necessarily correct, and I do not agree with them. I surely do not mean to remove the pulp with acids and arsenic and such things that will destroy the tooth, but if the pulp is removed surgically it does not destroy the tooth any more than removing the appendix destroys the whole alimentary canal; so that I indorse what Dr. Rhein says in respect to removing the pulp with rather more emphasis than perhaps he puts on it.

*Dr. N. S. Hoff,* Ann Arbor.—Would you have removed this pulp that Dr. Latham reports, simply because of the symptoms?

*Dr. Briggs.*—I think I would. It is one of my preliminary treatments in interstitial gingivitis or pyorrhœa alveolaris. I knew enough of the evils which are the result of this condition of the pulp before Dr. Latham and Dr. Talbot referred to the subject. I knew of the exostosis and the reverse action of the odontoblasts in removing calcareous matter from the root, and I knew of the pulp-stones, and now all these other conditions only emphasize, to my mind, the importance of the surgical removal of pulp when there is any irritation.

*Dr. M. L. Rhein.*—Dr. Briggs has stated my position, and I stand exactly on what I have published. Dr. Bogue has misunderstood what I have published. All that has ever been brought before this Section has shown us how difficult it is to find the pulps in adult life in a normal physiologic condition. As I understand Dr. Bogue, he advocates in the multiple-rooted teeth the use of arsenic in preference to removal under anæsthesia by cocaine. I differ entirely with him. I think he has held up the case which he cites a number of times, and very erroneously. The posterior root of this lower molar had coalesced with the anterior canals, and there was a slight amount of this deposit between the two roots. This is the description of the tooth given me by Dr. Taggart, of Burlington, Vt. After the pulp was surgically removed from the posterior canal and both anterior canals of these teeth were in such a condition that it was impossible to find a trace of any pulp-tissue, the canals were subjected to a most vigorous treatment by sodium and potassium, which practically destroyed any vitality that remained in this coalescent site. The subsequent history of this case proves absolutely the correctness of my position. It does not follow that arsenic would have acted any better. On the contrary, I have histories of cases in which arsenic has failed to do its work, and where cocaine enabled me to remove surgically the pulp from the canals of multiple-rooted teeth where it was impossible to proceed after numerous arsenical applications had been made. Where arsenic would have been of any further value in a case of coalescence of these two roots I fail to see.

*Dr. A. W. Harlan,* New York City.—It seems to me the summing up of Dr. Andrews's paper is the desirability of retaining the pulp in a tooth for other than formative purposes. Dr. Andrews's position on the retention of the vitality of the pulp when not diseased is probably correct. When a pulp becomes exposed there are certain changes that take place within a certain period which render that pulp as a normal organ valueless, and it might just as well be destroyed. Referring to the paper of Dr. Latham, I think perhaps the extraction of that tooth was correct, but I challenge the statement that there is no method of knowing whether the pulp has died, because we have ample means at present, although somewhat imperfect, of determining whether the pulp is alive.

*Dr. V. A. Latham.*—I said that our means of diagnosing dead teeth was not as yet very perfect or absolute.

*Dr. A. W. Harlan.*—So far as determining the vitality or nonvitality of the pulp is concerned, I should contend that with heat, cold, or electricity we can determine that absolutely. I was interested in Dr. Talbot's statement that abnormalities of the teeth occur from non-use. In the publication of Oakley Coles, more than thirty years ago, there are shown a number of abnormalities of the pulp. It is absolutely certain that when a tooth is not in use it deteriorates either by elongation or by the growths on the external surface of the root, or that it gets out of position and the tissues around the root become so inflamed and irritated that it becomes a useless member. It is further absolutely determined that in the rapid eruption of the teeth certain changes of the pulp take place. The chewing of tobacco and the holding of pipes in place also cause changes in the pulp. When the pulp is diseased we have a formation which may be in the shape of a cyst, and within that cyst we may have a deposit of calcareous matter which is a pseudo-form of tartar. There are growths around the ends of the teeth after the pulps die that may cause serious disturbance of the general system, so great as to result fatally. I would, therefore, conclude that when the pulp becomes exposed it should be destroyed, no matter whether it is surgically removed or poisoned; destroy it so that you will get every vestige of it out. The root of the tooth should be filled and there should be a sufficient amount of contact with the opposing jaw to keep the tooth in use.

*Dr. J. L. Williams,* Boston.—Pulps that are nearly exposed and not diseased I have proved in years of practice can be saved and means taken to protect them; but, after a pulp has become inflamed I have learned not to expect its total recovery. It may be kept comfortable, but I suppose its nutritive quality is lost, and I often question whether it is worth while to tamper with it. Some years ago I saw a case in which the pulp was exposed, but not wounded. I tried a process of treatment, which I was the first to introduce and systematize. I made the softest possible antiseptic covering for it; I looked at it at the end of a month and again treated it. At the end of about a year I opened it. It looked perfectly clear, and in passing my instrument over the transparent secondary dentine there was no sensation. It was then filled. In two years the tooth decayed on the other side. There was some sensation on excavating the new cavity, showing that we should discriminate between an exposed pulp and a diseased pulp. I do not believe that when a pulp has its growth it does not contribute to the continued welfare of the tooth. When it can be made healthy it should be saved for the benefit of the tooth.

*Dr. E. C. Briggs.*—We should bring out clearly that the idea brought forward is not that one is to go around slaughtering pulps of teeth, but that, if things are in any way wrong concerning the teeth, the chances are that there is more menace from the presence of the pulp than without it. In answer to Dr. Bogue, I do not like to have the principle destroyed by his questioning one's ability to remove the pulp. I do not pretend that I can do these things, but I think the chances are that I or some other one will find a perfect way of doing this work. We are doing now a great many things that could not be done ten years ago. The underlying principle is not affected.

*Dr. E. A. Bogue.*—Dr. Dawbarn, in a paper on malignant growths, speaks of an operation of his own devising,—viz., excision of a portion of both external carotids after ligation; and in speaking of that he has two or three times wondered why we as dentists did not more often observe the incipient beginnings of cancer. He says that sarcoma and carcinoma are acted on quite differently by his operation. In sarcoma he has had no instance up to the present in which he has felt that his operation has been a failure. In carcinoma he regards the operation as helpful only for a short time. That brought to mind Dr. Latham's case, which would show that it was through the circulation that carcinoma took its way, while sarcoma may more particularly be called a local condition. I hope we may have a further report concerning Dr. Latham's case.

*Dr. Talbot.*—There has been for several years a symposium on the dental pulp, and the results have been more than satisfactory. Dr. Andrews's paper is far-reaching, and I fear the importance of the paper is not understood. It has been discussed from the degeneration stand-point, but the fact has been lost sight of that the pulp at any period of life has its influence on tooth-structure. The paper is most valuable, because it leads to the point of the necessity of the vitality of the tooth. If it be necessary, as Dr. Andrews shows, it must follow that diseases of the human body have a great influence on tooth-structure. It must be admitted that there is a difference between supposition that certain conditions of the pulp exist, or that they have been found and actually demonstrated. Supposing a condition exists in the human body; to state it is one thing, to demonstrate it another. I wish all these papers on the pulp could be published in book form. It would be a most remarkable collection of papers.

*Dr. Latham.*—I came before this Section asking for a diagnosis, for advice, and also for bibliography. My main object was to bring my contribution before the Section for discussion as to the origin of the growth. I have held this case in hand for some three years, and only recently thought of publishing it. I have had experts in

Germany and in England looking up bibliography for me, with no success. The papers are not published in a way easy to be found, especially since the *Dental Cosmos* stopped its bibliography, and I would suggest greater care in choosing titles. I ask from what tissue did this epithelioma arise, if the pulp is a mesoblastic structure?

## Figures and Tables

**Fig. 1. f1:**
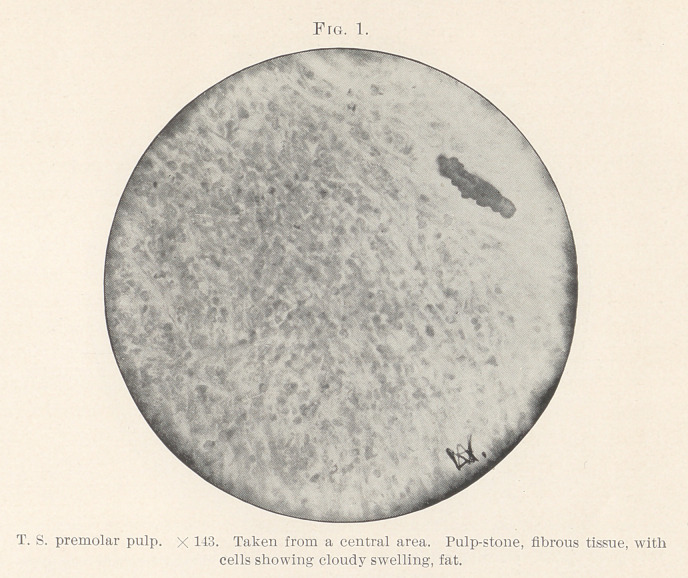


**Fig. 2. f2:**
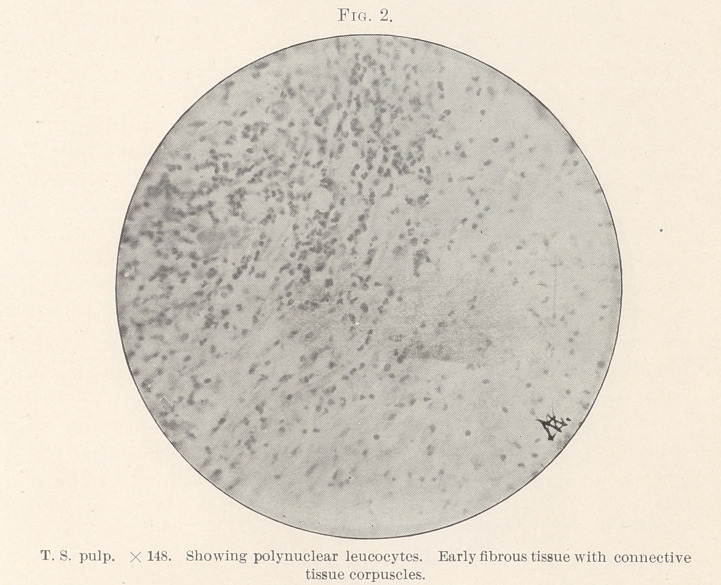


**Fig. 3. f3:**
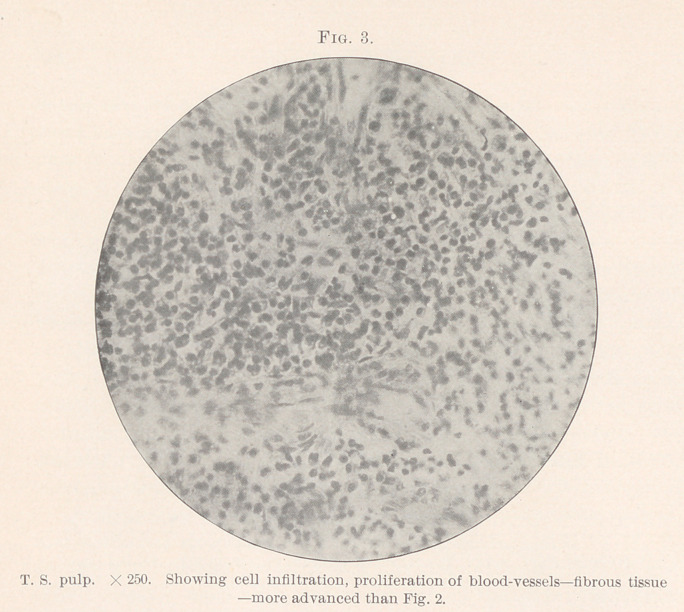


**Fig. 4. f4:**
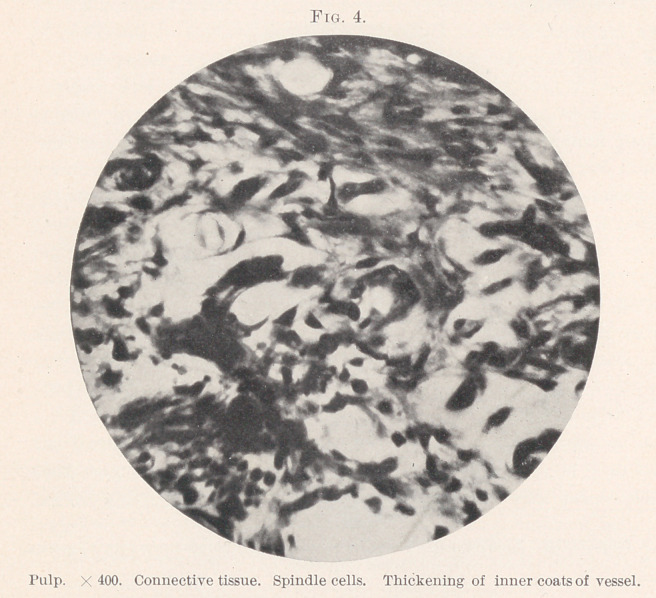


**Fig. 5. f5:**
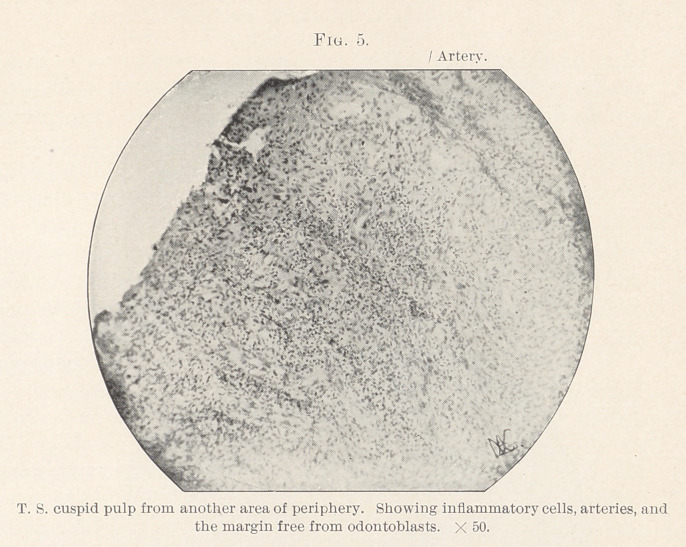


**Fig. 6. f6:**
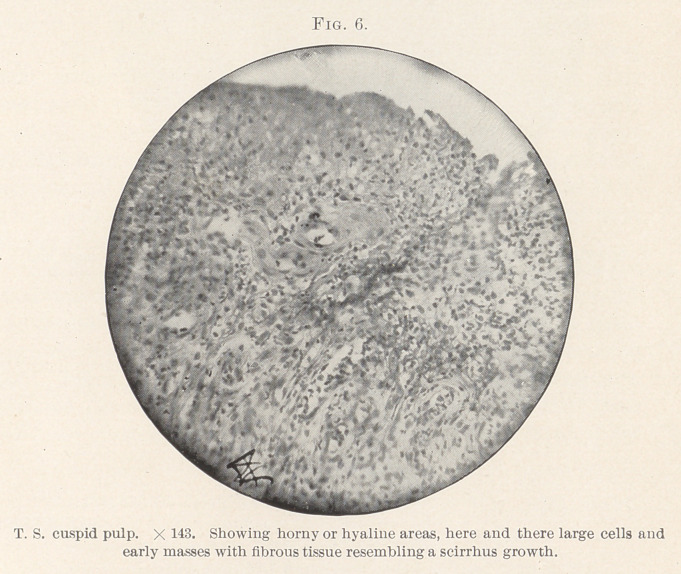


**Fig. 7. f7:**
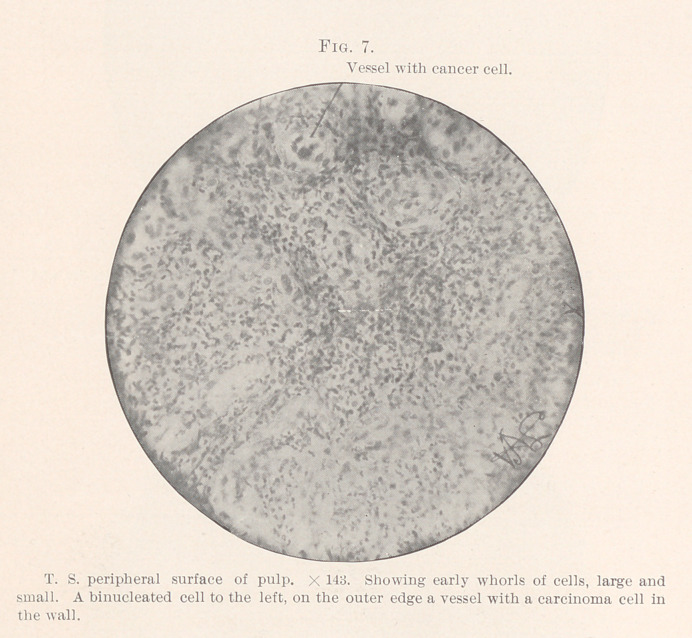


**Fig. 8. f8:**
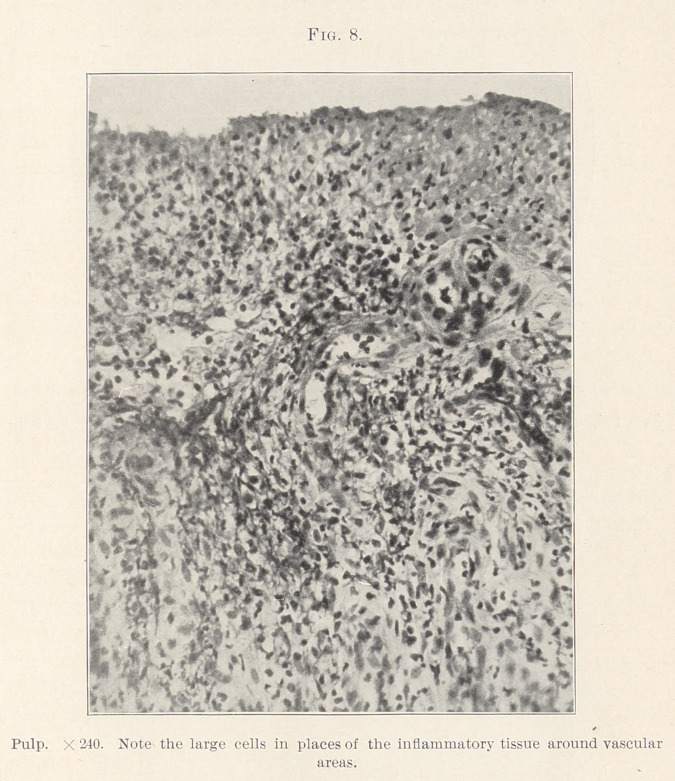


**Fig. 9. f9:**
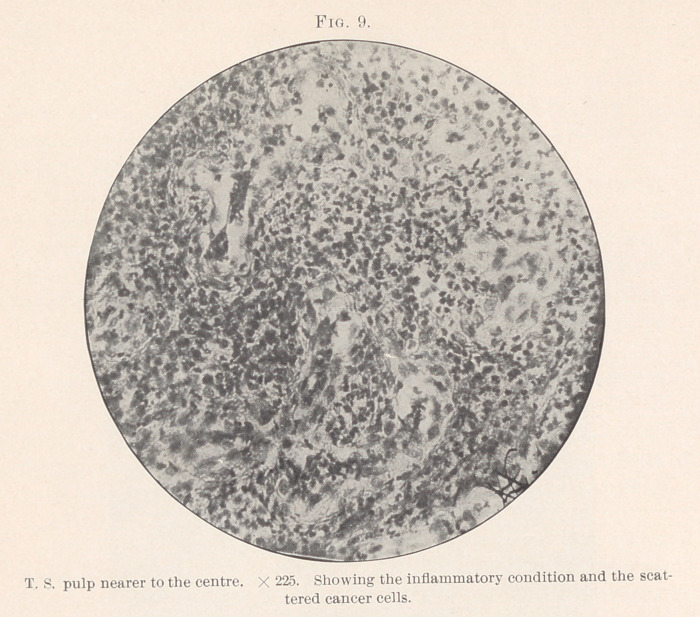


**Fig. 10. f10:**
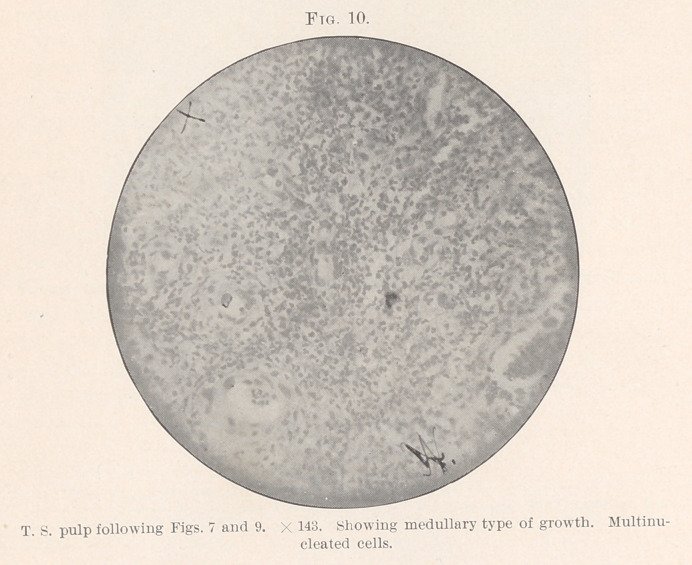


**Fig. 11. f11:**
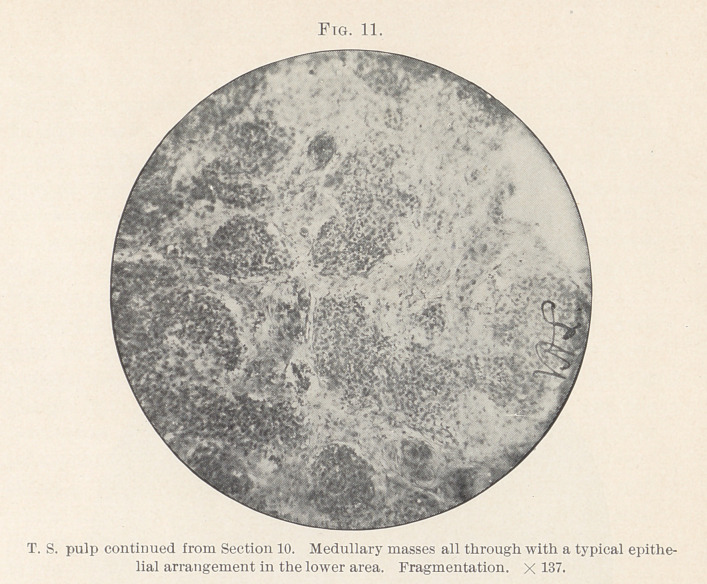


**Fig. 12. f12:**
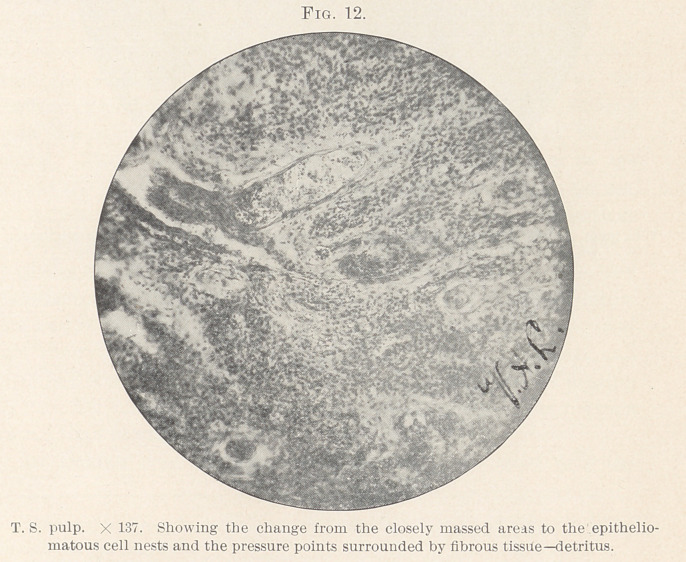


**Fig. 13. f13:**